# Sol–Gel Synthesis and Multi-Technique Characterization of Graphene-Modified Ca_2.95_Eu_0.05_Co_4_O_x_ Nanomaterials

**DOI:** 10.3390/polym17202767

**Published:** 2025-10-16

**Authors:** Serhat Koçyiğit

**Affiliations:** 1 Central Laboratory Application and Research Center, Bingöl University, 12000 Bingöl, Türkiye; sergas_29@hotmail.com or skocyigit@bingol.edu.tr; Tel.: +90-4262160012; 2 Institute of Science, Bingöl University, 12000 Bingöl, Türkiye

**Keywords:** graphene, nanomaterials, sol–gel, Scherrer equation

## Abstract

This study employs a multi-technique approach to elucidate how graphene incorporation affects phase formation, microstructure, and thermal behavior in PVA-assisted sol–gel synthesized Ca_2.95_Eu_0.05_Co_4_O_x_ nanomaterials. XRD confirms the preservation of the primary phases (hexagonal CaCO_3_ and cubic CoO) alongside a distinct graphene (002) reflection; a systematic low-angle shift of the calcite (104) peak evidences partial relaxation of residual lattice strain with increasing graphene content, while Scherrer analysis indicates tunable crystallite size. Raman spectroscopy corroborates graphene incorporation through pronounced D (~1300 cm^−1^) and G (~1580 cm^−1^) bands and supports the XRD-identified phase coexistence via cobalt-oxide and calcite vibrations in the 200–700 cm^−1^ region, also indicating increased defect/disorder with graphene loading. SEM shows grain refinement, denser/bridged lamellar textures, and reduced porosity at low–moderate graphene contents (1–3 wt.%), contrasted by agglomeration-driven heterogeneity at higher loadings (5–7 wt.%). EDX reveals increasing carbon with Ca/Co redistribution at accessible surfaces, and TG–DSC corroborates the removal of oxygen-containing groups and oxidative combustion of graphene at mid temperatures. Collectively, Raman–XRD-consistent evidence demonstrates that graphene provides a tunable handle over lattice strain, crystallite size, and grain-boundary architecture, establishing a processing–composition basis for optimizing functional (e.g., electrical/thermoelectric) performance.

## 1. Introduction

Contemporary technological progress is strongly oriented toward miniaturization and the maximization of functional surface area [1]. Reducing the dimensions of devices and materials affords key advantages such as lower raw material consumption, enhanced efficiency, portability, and the integration of multiple functionalities [2,3]. In this context, nanotechnology-enabled manufacturing routes provide a powerful platform for developing next-generation high-performance materials [4]. Post synthesis strategies—including targeted doping, surface functionalization, and nanofiller incorporation—enable the purposeful optimization of mechanical, thermal, optical, and chemical durability characteristics [5,6].

Among the synthesis routes for nanostructured materials, the sol–gel process stands out owing to its relatively low processing temperatures, precise stoichiometric control, chemical homogeneity, compatibility with multicomponent systems, tunable morphology, and scalability [7,8]. During sol–gel processing, metal (or metalloid) alkoxides or metal salts are transformed into hybrid nanomaterials with polymeric matrices through physical or chemical crosslinking mechanisms [9].

Nanomaterials produced by these methods exhibit high specific surface area, adjustable porosity, improved mechanical strength, and superior thermal and/or chemical stability, conferring industrial applicability across energy, materials, defense, biological, and chemical sectors [10,11]. Within advanced nanotechnological materials, calcium cobalt oxide structures possess broad utility in ceramic, energy storage, and thermoelectric applications due to their high temperature stability, favorable electrical conductivity and catalytic activity at elevated temperatures, and intrinsically low thermal conductivity [12,13,14]. Low level doping with rare earth elements such as europium, cerium, or praseodymium can induce controlled modifications in morphological architecture, thereby tailoring surface area, particle size, and porosity to elevate overall material performance [15,16].

Graphene, owing to its single-atom thickness and two-dimensional (2D) crystalline architecture, offers extremely high in-plane carrier mobility, which in turn imparts outstanding electrical as well as mechanical performance to graphene-modified systems [17,18]. These salient features mean that incorporating graphene as a dopant/additive into a host matrix can confer pronounced functional enhancements. Graphene is introduced as a microstructural regulator to promote homogeneous dispersion, mitigate agglomeration, and refine crystallite-size distribution within the CaCO3–CoO precursor.

This study aims to systematically elucidate the effect of graphene incorporation—an aspect only sparsely addressed in the literature—on the microstructural evolution of PVA-assisted Eu-doped Ca–Co–O sol–gel precursors. Our hypothesis is that uniformly dispersed graphene sheets modulate the crystallite size of the doped parent matrix, thereby identifying a tunable pathway for optimizing the microstructure toward improved performance in subsequent functional products.

## 2. Materials and Methods

Calcium, europium, and cobalt oxide precursors were introduced in the form of their acetate salts. Europium(III) acetate hydrate (99.9%, CAS: 62667-64-5), calcium acetate hydrate (≥99.0%, CAS: 114460-21-8), and cobalt(II) acetate tetrahydrate (≥98.0%, CAS: 6147-53-1) were procured from Sigma Aldrich, Darmstadt, Germany. The graphene source (nano-synthesized graphene, nominal lateral size 4–6 nm) was obtained from Grafen Co. Ind, Ankara, Türkiye. Deionized water and glacial acetic acid (Merck, Darmstadt, Germany) were used to prepare the salt solutions. Poly(vinyl alcohol) (PVA, Mw 85,000–124,000) supplied by Sigma Aldrich (in Darmstadt, Germany) was employed as the polymer precursor. Five nanomaterial compositions were synthesized and were coded TS1–TS5 to distinguish incremental graphene additions to the Ca_2.95_Eu_0.05_Co_4_O_x_ host system. TS1 denoted the graphene-undoped baseline, while TS2–TS5 denoted graphene-doped nanomaterials at progressively higher graphene loadings. Accordingly, the sample designations were as follows:

- TS1: graphene-undoped Ca_2.95_Eu_0.05_Co_4_O_x_ nanomaterials (0 wt.% graphene).
- TS2: 1% graphene-doped Ca_2.95_Eu_0.05_Co_4_O_x_ nanomaterials (1 wt.% graphene).
- TS3: 3% graphene-doped Ca_2.95_Eu_0.05_Co_4_O_x_ nanomaterials (3 wt.% graphene).
- TS4: 5% graphene-doped Ca_2.95_Eu_0.05_Co_4_O_x_ nanomaterials (5 wt.% graphene).
- TS5: 7% graphene-doped Ca_2.95_Eu_0.05_Co_4_O_x_ nanomaterials (7 wt.% graphene).

During sol–gel, acetate-based metal ions undergo hydrolysis followed by condensation to build M–O–M bridges, assisted by PVA and acetic acid:

Hydrolysis:

M–OR + H_2_O → M–OH + ROH

Condensation:

(a) M–OH + HO–M → M–O–M + H_2_O

(b) M–OH + RO–M → M–O–M + ROH

Here, M = Ca, Co, and Eu. PVA/acetic acid aid complexation and pH buffering improve sol stability; calcination removes organics to yield an oxide network.

In the first step of synthesis (solution formation), the calcium, europium, and cobalt acetate salts (with the proportions as 2.95:0.05:1.00, respectively) were magnetically stirred in deionized water with acetic acid for 6 h at 300 rpm at room temperature. For gel preparation, a 10% (*w*/*v*) PVA solution was prepared by dissolving PVA in deionized water at 80 °C under stirring for 2 h, then allowed to cool to room temperature. The PVA solution was subsequently added to the calcium, europium, and cobalt acetate solution, and the mixture was maintained on a magnetic stirrer at 300 rpm, 40 °C for 12 h, yielding the sol–gel solution. The amounts of calcium, europium, and cobalt salts and PVA solution were given in Table 1. To induce gelation, the solutions were transferred into porcelain crucibles and kept in a drying oven at 100 °C for 24 h. The resulting xerogels were then calcined in a tubular furnace at 500 °C to promote oxide formation and remove most organics. The obtained oxide powders were gently ground in an agate mortar, pressed into pellets (8 mm diameter, ~3 mm thickness) under 6 tons uniaxial load using a hydraulic press, and sintered at 650 °C for 4 h in a tubular furnace to achieve consolidation. The sintered pellets were characterized by XRD, SEM, FTIR, Raman, and simultaneous thermal analysis (STA).

Structural and morphological characterizations were conducted using several analytical techniques. X-ray diffraction (XRD) patterns were collected on a PANalytical Empyrean diffractometer over 2θ = 10–70°, with Cu Kα radiation (λ = 1.5406 Å), Bragg–Brentano θ–2θ geometry, a Ni filter/monochromator, a step size of 0.02° 2θ, and a scan rate of ≈2°/min. Powders were mounted on a low-background holder, surface leveled; gentle pressing was applied to limit the preferred orientation. Fourier Transform Infrared (FTIR) spectra were recorded with a PerkinElmer Spectrum 100 spectrometer. Scanning Electron Microscopy (SEM) images were obtained using a JEOL JSM-6060 microscope. Powders were drop-cast onto Si wafer/alumina stubs. To mitigate charging, samples were DC sputter-coated with a 5–8 nm Au/Pd layer. SEM was conducted at a 20 kV accelerating voltage and a 5–8 mm working distance. EDS was performed for point/area compositional analysis and elemental mapping (Ca, Co, O, and trace Eu distribution). Thermal (TG-DSC) measurements were performed on a Setaram Labsys Evo simultaneous thermal analysis system. Raman spectroscopy was performed to analyze the structural properties of the nanomaterials using a Renishaw Centrus 2945K3 detector with a 785 nm laser (0.01% power) and a 1200 L/mm grating, under a 50× objective. To optimize the signal-to-noise ratio, an exposure time of 30 s with 10 accumulations was employed, and spectra were recorded over the Raman shift range of 100–4000 cm^−1^.

## 3. Results and Discussion

### 3.1. Fourier Transform Infrared Spectroscopy (FT-IR) Results

**Figure 1:**
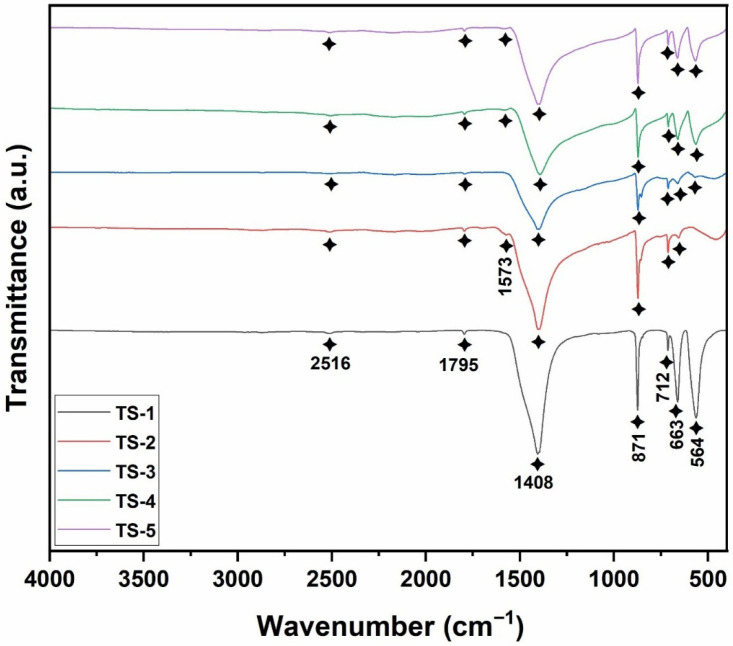
FT-IR spectra of TS-1–5 nanomaterials.

In the FTIR spectrum given in Figure 1, bands at 1795, 871, and 712 cm^−1^ represented characteristic carbonate ion vibrations associated with the calcium carbonate lattice [1]. Additionally, the bands at 1408 and 871 cm^−1^ corresponded to the symmetric stretching (ν1) and out-of-plane bending (ν2) modes of carbonate ions, respectively [19]. In dolomitic structures, in-plane bending modes of carbonate groups appeared at approximately 712 cm^−1^ [20]. Distinct peaks at 564 and 663 cm^−1^ confirmed the spinel-type structural features of cobalt oxide nanoparticles [21]. The band at 1573 cm^−1^ evidenced the presence of graphene, and its appearance in all samples except TS-1 corroborated graphene incorporation [22]. Literature indicated that, due to the complexity of overlapping bands below 900 cm^−1^, the assessment of graphene presence was preferably based on bands above this region [23]. The collected FTIR data therefore substantiated the coexistence of calcium carbonate, cobalt oxide, and graphene components within the nano-structure. The europium signal was not discernible in FTIR, which was attributed to its very low doping level.

### 3.2. X-Ray Diffraction (XRD) Results

**Figure 2:**
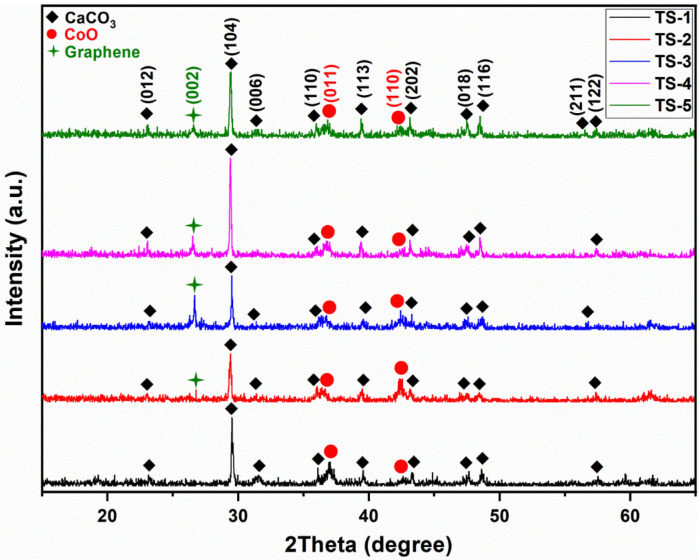
XRD patterns of TS-1–5 nanomaterials.

The XRD patterns of samples TS-1 through TS-5 were presented in Figure 2. Analysis revealed hexagonal CaCO_3_ and cubic CoO phases [24]. The CaCO_3_ reflections matched ICDD card 98-016-4935 and the CoO reflections matched ICDD card 98-017-4027. No distinct europium-related diffraction peak was detected, consistent with its low concentration; however, europium was confirmed by EDX and elemental mapping. Across the TS-1–TS-5 series, the primary CaCO_3_–CoO phase assemblage was preserved. A (002) graphene reflection appeared near 2θ ≈ 26.50° in graphene-containing samples TS-2 through TS-5 [25]. The presence of the 1573 cm^−1^ band in FTIR and the decomposition features of oxygen-functional groups in mid-temperature TG–DSC profiles further supported graphene incorporation.

Graphene, rather than incorporating into the host lattice at the ionic/atomic level, is distributed along grain boundaries as an interfacial additive. The presence of the graphene (002) reflection in XRD indicates that graphene exists as a separate carbon phase. Therefore, its integration into the structure occurs not via chemical substitution but through interfacial/nanostructural incorporation along grain boundaries.

For the determination of hexagonal CaCO_3_ lattice parameters, the following relation was employed [26]:

(1) $$\frac{1}{d^{2}}=\frac{1}{\left(4/3a^{2}\right)\left(h^{2}+k^{2}+hk\right)+(l^{2}/c^{2})}$$

where *d* was the interplanar spacing, (*hkl*) were Miller indices, and *a* and *c* were the hexagonal lattice constants.

To estimate crystallite size for the principal CaCO_3_ reflection, the full width at half maximum (FWHM) of the main peak in the XRD diffractogram was extracted (OriginPro 8.5). The Debye–Scherrer equation was then applied [27]:

(2) $$D_{hkl}=\frac{K\cdot \lambda }{\beta_{hkl}cos\theta }$$

where $D_{hkl}$ was the average crystallite size, *K* = 0.9 was the shape factor, *λ* = 0.1540562 nm (Cu Kα_1_), $\beta_{hkl}$ was the FWHM (in radians) of the selected peak, and *θ* was the Bragg angle.

Lattice parameters computed from Equations (1) and (2) for the hexagonal CaCO_3_ phase are summarized in Table 2. These were derived solely from the (104) reflection, which exhibited the highest fitting reliability (best R^2^) relative to other reflections in the pattern. The (104) peak occurred at 2θ = 29.52° for TS-1, while in the remaining samples it was predominantly located at 29.39°. This ~0.13° shift toward a lower angle corresponded, via Bragg’s law, to a slight increase in d spacing and explained the apparent expansion in a (4.9700 → 4.9915 Å) and c (16.9925 → 17.0660 Å). After graphene addition, the peak position moved closer to the standard calcite values listed in ICDD 98 016 4935. By analogy with the report of Ahmad et al. (2014) on Mn-doped ZnO, where dopant ionic radius mismatch induced strain and slight peak shifts ((101) reflection shifting to a lower angle), the present behavior was interpreted as strain relaxation; here, the source was attributed not to a Zn–Mn disparity but to partial incorporation tendencies of smaller Co^2+^ (and possibly charge compensating Eu^3+^) within residual CaCO_3_ domains [28].

The FWHM values (0.1418°, 0.2059°, 0.1342°, 0.1172°, and 0.1626°) showed a non-monotonic profile, and the corresponding Scherrer crystallite sizes (58.69, 40.40, 62.01, 70.99, and 51.16 nm) varied accordingly. At low graphene addition (TS-2), peak broadening led to a reduced crystallite size; in TS-3 and TS-4, peak narrowing yielded larger crystallites; and in TS-5, a renewed broadening reduced size again. In TS-2 spectrum (low loading, broad peak), broad Bragg peaks indicate small Scherrer crystallite size and relatively high microstrain. At low graphene loading, sheet distribution to interfaces is expected, but the formation of a percolative continuity is uncertain; the broader, moderate-intensity TG–DSC exotherm and the partial inhomogeneity in SEM support this [29,30]. In TS-3 and TS-4 spectra (intermediate loading, narrow peak), graphene is better dispersed and forms a percolative network that balances heat and oxygen access [29]. The sharp, single-step TG–DSC exotherm and the homogeneous microstructure in SEM indicate more uniform sintering. In TS-5 spectrum (high loading, re-broadened peak), excess graphene leads to agglomeration and local diffusion constraints [31,32]. The shouldered, broadened TG–DSC exotherm signals multi-stage combustion and a heterogeneous thermal state, which in turn suppresses grain growth in some regions while promoting irregular recrystallization in others. In TS-1, the smaller ionic radius of Co^2+^ compared to Ca^2+^ was considered to have imposed a net lattice contraction (peak at higher 2θ) within carbonate domains. In graphene-containing samples, graphene facilitated more effective transfer of Co into the primary layered Ca–Co oxide phase, enriching the carbonate residue in Ca and thereby reducing residual strain. Consequently, graphene addition decreased the magnitude of strain-related peak displacement rather than suppressing peaks entirely.

### 3.3. Scanning Electron Microscopy (SEM) Images

**Figure 3:**
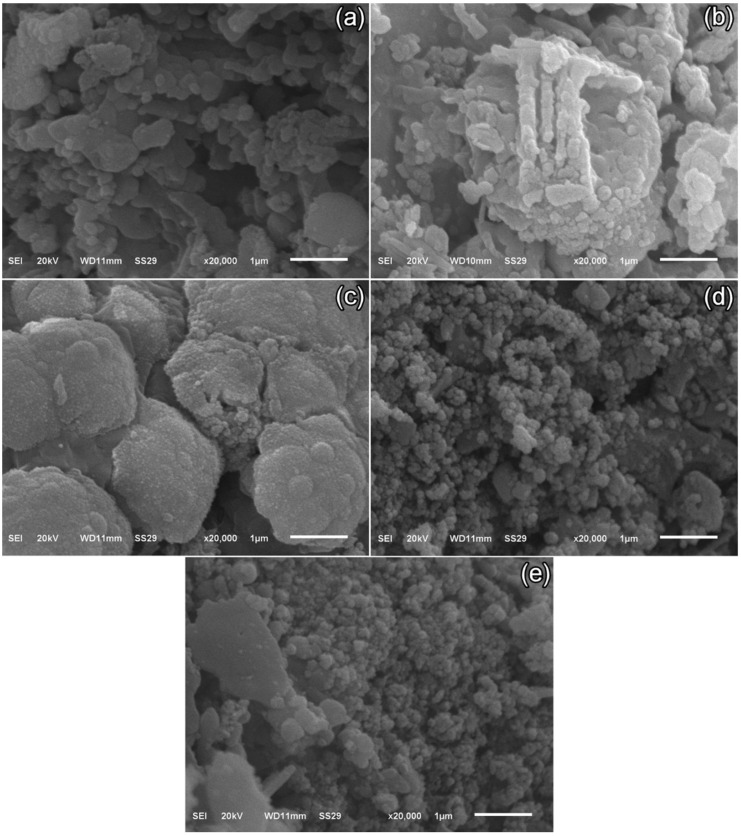
SEM images of the (**a**) TS-1, (**b**) TS-2, (**c**) TS-3, (**d**) TS-4, and (**e**) TS-5 samples at 20,000× magnification.

SEM micrographs of TS-1–TS-5 (Figure 3a–e) were acquired at 20 kV, spot size 29, and 20,000× magnification. These images elucidated microstructural evolution with varying graphene content in the Ca_2.95_Eu_0.05_Co_4_O_x_ nanomaterial. In graphene-free TS-1, a platelet (lamellar/flaky) morphology predominated, with irregular edges, partial stacking, and open porosity. In TS-2 (1% graphene), finely dispersed graphene sheets enveloped grain boundaries, refining average platelet size to ~200–350 nm and producing a denser microstructure. TS-3 (3% graphene) exhibited enhanced intergranular bridging by the expanded graphene network; platelets appeared more fragmented/truncated, reaching ~150–250 nm with narrower pores. In TS-4 (5% graphene), localized graphene agglomeration induced microstructural heterogeneity: very fine (~150–200 nm) grains persisted where pinning was active, whereas regions around agglomerates exhibited regrown platelets of 180–400 nm (occasionally >500 nm) due to local heat transfer and sintering disparities. In TS-5 (7% graphene), excessive loading yielded pronounced dispersion alongside agglomerates; nano-platelet clusters of ~100–150 nm formed. Overall, low to moderate graphene levels (1–3%) suppressed grain coarsening and fostered a finer, more uniformly bonded microstructure, while higher additions (5–7%) promoted agglomeration-driven heterogeneity.

At low graphene loadings (TS-2–TS-3), microstructural connectivity improved beyond mere size effects, with enhanced intergranular bridging and interfacial continuity; this was reflected in a more uniformly distributed population of open pores. Concurrently, locally aligned platelet stacks indicated the emergence of qualitative texturing that may have facilitated the formation of continuous conductive pathways. In contrast, at higher loadings (TS-4–TS-5), agglomerate parts were accompanied by anisotropic, irregular pores, indicative of disrupted capillary transport. This behavior aligned with reports that well-dispersed graphene at low contents refined porosity via interfacial bridging, thereby strengthening intergranular coupling and maintaining the continuity of heat/electrical transport, whereas excessive graphene fostered agglomeration, pore anisotropy, and textural heterogeneity that undermined densification and percolation [33,34,35,36].

### 3.4. Energy-Dispersive X-Ray Spectroscopy (EDX) Results

Elemental compositions (wt.%) for TS-1–TS-5 are summarized in Table 3 with corresponding mapping images provided in the Supplementary Information (Figure S1). Carbon content increased from 6.080 wt.% in graphene-free TS-1 to 10–21 wt.% in graphene-containing samples, reflecting both carbonate and added graphene contributions (TS-1 carbon arose mainly from carbonate). Although higher carbon percentages nominally diluted metal cation weight fractions through normalization, fluctuations in Ca, Co, and Eu exceeded simple dilution effects, indicating genuine redistribution. Cobalt content initially decreased at low graphene addition, then rose sharply with increased graphene (TS-3–TS-4), before declining again at the highest loading (TS-5). This was attributed to cobalt-enriched platelet surfaces in TS-3–TS-4 and reduced surface accessibility plus agglomeration effects in TS-5. Calcium content showed an initial dilution followed by partial recovery, exhibiting an inverse (phase-counterbalancing) trend relative to cobalt. Europium remained within 0.24–0.79 wt.% across samples; its low absolute concentration and local inhomogeneity, combined with near-surface interaction volume constraints, explained the small variations. Elemental maps confirmed Eu distribution patterns and validated the EDX quantification.

### 3.5. Thermogravimetric Analysis (TGA) Results

**Figure 4:**
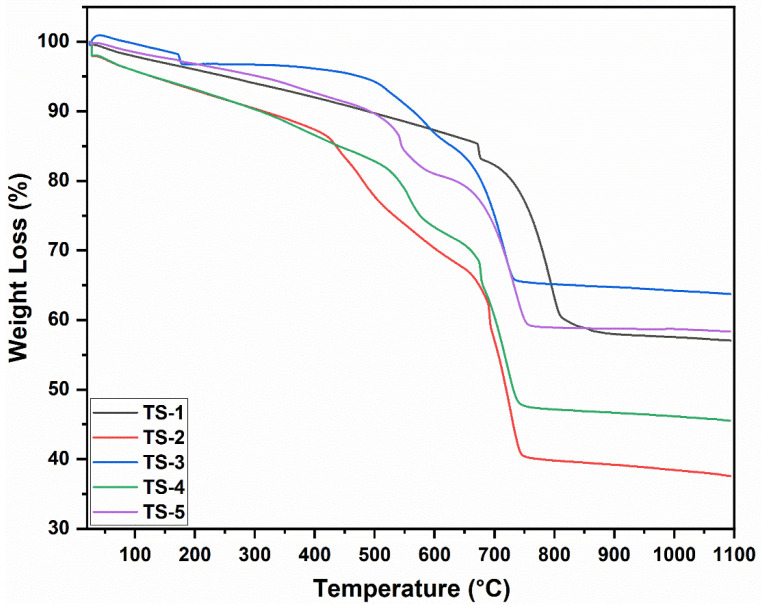
TGA curves of TS-1–5 nanomaterials.

The TGA curves in Figure 4 showed that graphene-free TS-1 exhibited the lowest mass loss, limited to the desorption of moisture and trace organics. With graphene addition, a two-stage enhancement emerged: (i) 150–350 °C—the decomposition of oxygen-functional groups and residual organics; (ii) 350–550 °C—the principal oxidation/combustion of the graphene carbon framework. At intermediate graphene loadings (TS-3–TS-4), the main combustion step became sharper, indicating improved dispersion (homogeneous oxidative access). At the highest loading (TS-5), the decomposition profile broadened, with diffusion-limited, multi-shoulder characteristics consistent with graphene agglomeration. Minimal further mass change above ~600 °C evidenced the thermal stability of the oxide matrix and near-complete consumption of labile carbon at mid temperatures. A slight slope softening between 700 and 900 °C was related to partial oxygen vacancy refilling or subtle redox equilibration. Although cumulative mass loss trends broadly tracked nominal graphene content, the modest difference between TS-4 and TS-5 supported the notion that agglomeration reduced effective combustible surface area. The collective thermal behavior coherently aligned with EDX-reported heterogeneous carbon distribution and SEM-identified flake/agglomerate morphologies. The inclusion of derivative (DTG) peak temperatures in the manuscript would have strengthened correlations among graphene dispersion quality, oxygen stoichiometry, and prospective electrical/thermoelectric performance.

### 3.6. Differential Scanning Calorimetry Analysis (DSC) Results

**Figure 5:**
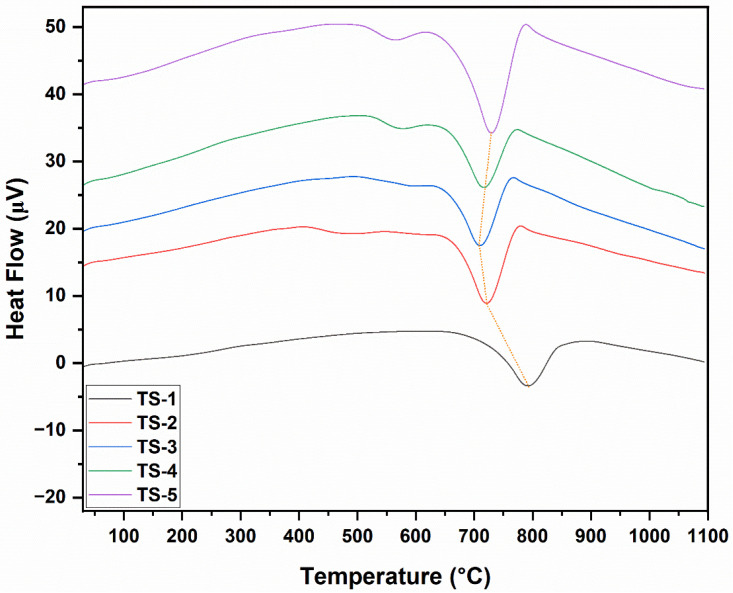
DSC graphs of TS-1–5 nanomaterials.

The DSC graphs of TS-1–5 samples are given in Figure 5. DSC heat-flow profiles corroborated the TGA mass-loss steps. A narrow, low-intensity endothermic signal at ≈40–140 °C appeared in all samples and corresponded to the desorption of physisorbed water (TGA’s initial minor loss). A subtle shoulder or broad low-intensity endothermic to mild exothermic transition at 150–320 °C was ascribed to the removal of oxygenated functional groups on graphene, residual organics, and partial surface oxidation pre-activation (second TGA segment). The principal exothermic peak at 330–560 °C, whose area increased with graphene loading, reflected the dominant carbon (graphene) oxidation step observed in TGA; it was weak in TS-1, became stronger and more defined in TS-2–TS-4, and broadened with possible shoulder development in TS-5 due to diffusion-limited multi-stage combustion from agglomeration. A slight elevation in the peak maximum temperature at intermediate loading suggested improved dispersion and optimized heat/oxygen transfer across the matrix–graphene interface, whereas diminished definition at high loading signified reduced effective reactive surface. The absence of major new exothermic events beyond 600 °C was consistent with mass stabilization and confirmed residual phase robustness. A minor baseline drift (if present) between 700 and 900 °C was associated with low-enthalpy rearrangements or partial oxygen vacancy reoccupation. In particular, the DSC profiles of TS-2–5 samples, especially the exothermic peak in the 550–650 °C range, revealed an oxidative combustion signature that was sensitive to the accessibility of the carbon phase. In TS-3–TS-4 samples, the peak became sharper and more single-step in character, indicating more homogeneous graphene dispersion and activated reactive surface area. In the TS-5 sample, the peak broadened and developed a shoulder, consistent with agglomeration that locally limits oxygen diffusion. In the TS-2 sample, a moderate-intensity, relatively broad feature was observed. This suggested that at low loading, the graphene sheets had spread to the surfaces/interfaces but had not yet formed a fully percolative continuity. These trends were consistent with literature findings. During sintering, well-dispersed graphene at intermediate doping levels (TS-3 and TS-4) had enhanced heat and oxygen access and concentrated the combustion within a narrow temperature window. In contrast, at high doping levels (TS-5), agglomeration had kinetically heterogenized the combustion process [33].

### 3.7. Raman Results

**Figure 6:**
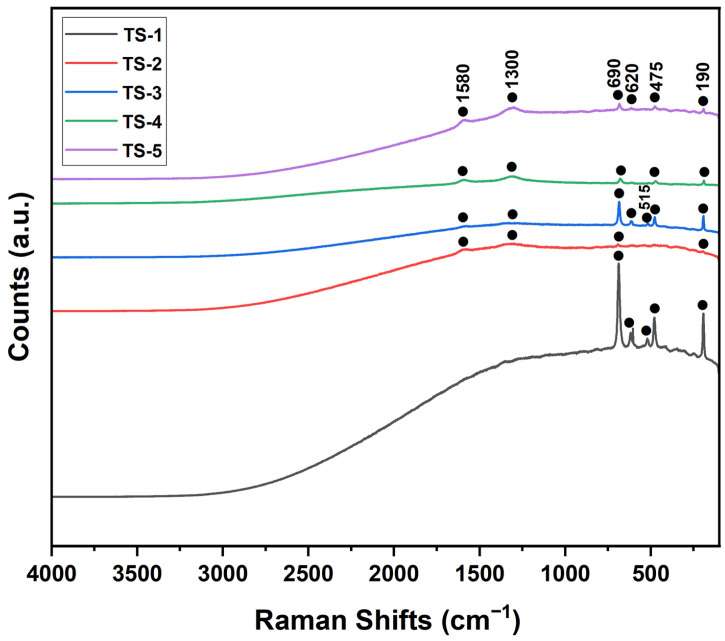
Raman spectra of TS-1–5 nanomaterials.

Raman spectra for samples TS-1 through TS-5 were presented in Figure 6. All spectra exhibited distinct vibrational features at approximately 190, 475, 620, and 690 cm^−1^, whereas the band near 515 cm^−1^ was detected only in TS-1 and TS-3. In TS-2–TS-5, additional bands emerged at ~1300 and ~1580 cm^−1^ that were absent in TS-1, indicating the presence of a graphene phase. Based on standard assignments, the 475, 515, and 620 cm^−1^ bands were associated with cobalt-oxide vibrations, while the 190 and 690 cm^−1^ bands were attributed to the calcium carbonate phase [37]. In the graphene-containing samples (TS-2–TS-5), the D band (~1300 cm^−1^), indicative of structural defects/disorder, and the G band (~1580 cm^−1^), corresponding to in-plane vibrations of sp^2^-hybridized carbon, were clearly resolved. The intensities of these graphene-related bands increased progressively across the series: they were weakly discernible in TS-2 and TS-3, became more pronounced in TS-4, and were strongly amplified in TS-5, reflecting higher graphene loading. This trend was consistent with the graphene (002) reflection observed by XRD and demonstrated that increasing graphene content enhanced structural disorder and the spectroscopic contribution of the carbon phase.

## 4. Conclusions

Overall, the combined spectroscopic, diffraction, microscopic, compositional, and thermal analyses established that controlled graphene incorporation modulated lattice strain, crystallite refinement, grain boundary evolution, and carbonaceous phase dispersion—factors expected to influence the functional (e.g., electrical/thermoelectric) performance of the Ca_2.95_Eu_0.05_Co_4_O_x_-based nanomaterials.

The data confirmed that maintaining graphene at optimal levels was critical: the sharper, single-step TG–DSC exothermic peak observed in the 550–650 °C range for TS3–TS4 indicated more homogeneous graphene dispersion, the activation of reactive surface area, and enhanced heat/oxygen accessibility that concentrated combustion within a narrow temperature window, and this interpretation was further supported by SEM results, which corroborated both the suppression of agglomeration and the improved structural homogeneity.

The Ca–Co–O-based systems investigated in this study and their graphene-modified derivatives offer potential applications enabled by grain-boundary engineering, interfacial continuity, and the controlled distribution of the carbon phase, including thermoelectrics (high-temperature phase stability and low thermal conductivity for waste-heat recovery and industrial power generation, with graphene interfaces improving electrical conductivity, contact resistance, and power factor), energy storage in supercapacitor electrodes (where 2D Ca–Co oxide architectures combined with a conductive/porous graphene network provide advantages), and device-level integration in ceramic-based thermoelectric modules and high-temperature sensors (reduced contact resistance and improved electrode–material compatibility). To substantiate these prospects, future work will focus on direct interfacial visualization via HRTEM/SAED, strengthening correlations through electrical transport measurements (Seebeck coefficient, electrical conductivity, and power factor), and integrating electrode architectures using scalable fabrication methods, which is expected to provide significant contributions to the literature.

## Figures and Tables

**Table 1:** Amount of the sol–gel solution components.

Solution #	Amount of Components (g)				
	Calcium Acetate	Europium Acetate	Cobalt Acetate	Graphene	PVA Solution (10%)
TS-1	2.5936	0.0216	3.3812	-	15
TS-2	2.5936	0.0216	3.3812	0.0150	15
TS-3	2.5936	0.0216	3.3812	0.0450	15
TS-4	2.5936	0.0216	3.3812	0.0750	15
TS-5	2.5936	0.0216	3.3812	0.1050	15

**Table 2:** Lattice parameters of the unit cells (hexagonal CaCO_3_ phase).

Sample	Crystal Structure	*2θ* (°)	FWHM (°)	Peak Intensity (a.u.)	(*hkl*)	*a* (Å)	*c* (Å)	*d* (Å)	*D_hkl_* (nm)
TS-1	*h*-CaCO_3_	29.52	0.1418	50.66	(104)	4.9700	16.9925	3.02350	58.69
TS-2	*h*-CaCO_3_	29.39	0.2059	30.87	(104)	4.9915	17.0660	3.03658	40.40
TS-3	*h*-CaCO_3_	29.49	0.1342	39.37	(104)	4.9750	17.0094	3.02651	62.01
TS-4	*h*-CaCO_3_	29.39	0.1172	76.79	(104)	4.9915	17.0660	3.03658	70.99
TS-5	*h*-CaCO_3_	29.39	0.1626	51.21	(104)	4.9915	17.0660	3.03658	51.16

**Table 3:** EDX composition values of TS-1–5 nanomaterials.

Element	Line	TS-1	TS-2	TS-3	TS-4	TS-5
		Conc (wt.%)	Conc (wt.%)	Conc (wt.%)	Conc (wt.%)	Conc (wt.%)
Carbon (C)	Ka	6.080	10.792	18.801	21.521	20.916
Oxygen (O)	Ka	40.806	49.088	32.118	21.881	39.340
Calcium (Ca)	Ka	38.541	30.528	17.139	24.753	22.133
Cobalt (Co)	Ka	13.969	9.351	31.202	31.059	17.077
Europium (Eu)	La	0.604	0.240	0.740	0.786	0.533
**Total**		**100.000**	**100.000**	**100.000**	**100.000**	**100.000**

## Data Availability

The original contributions presented in this study are included in the article. Further inquiries can be directed to the author.

## Supplementary Materials

The following supporting information can be downloaded at https://www.mdpi.com/article/10.3390/polym17202767/s1. Figure S1: The mapping images of TS-1–5 nanomaterials.

## Abbreviations

The following abbreviations are used in this manuscript:

FTIR	Fourier Transform Infrared Spectroscopy
SEM	Scanning Electron Microscope
XRD	X-Ray Diffractometer
EDX	Energy-dispersive X-ray Spectroscopy
TGA	Thermogravimetric Analysis
DSC	Differential Scanning Calorimetry
FWHM	Full Width at Half Maximum
ICDD	The International Centre for Diffraction Data
