# Supplementary figures and images for: Sol–Gel Synthesis and Multi-Technique Characterization of Graphene-Modified Ca2.95Eu0.05Co4Ox Nanomaterials
Source: Polymers (Basel). 2025 Oct 16;17(20):2767. doi: 10.3390/polym17202767 (PMC12567225; doi:10.3390/polym17202767)

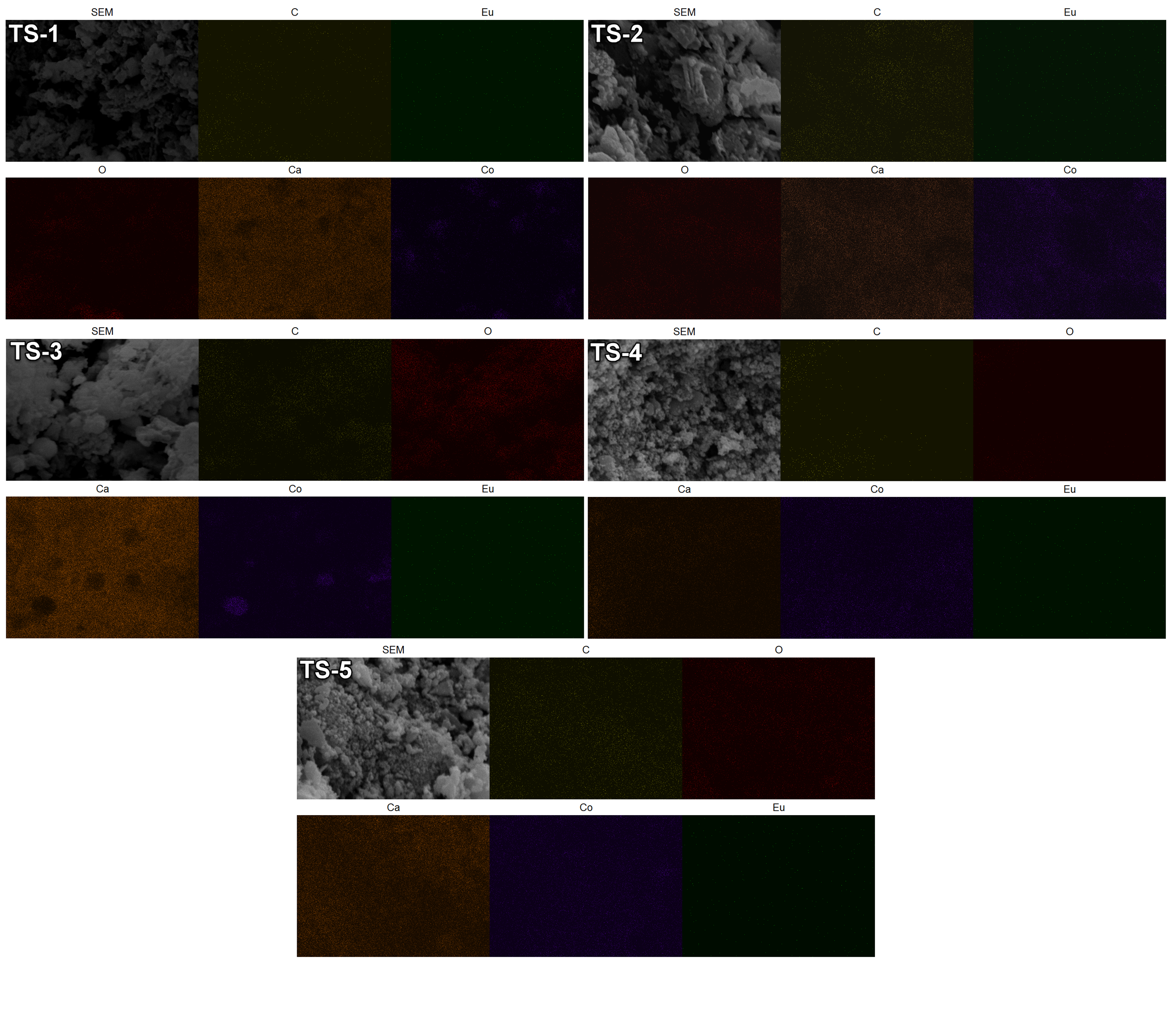

Supplement: Supplementary file 1 [file polymers-17-02767-s001.zip › polymers-3876621-supplementary.tif]
